# Gallic and Hesperidin Ameliorate Electrolyte Imbalances in AlCl_3_-Induced Nephrotoxicity in Wistar Rats

**DOI:** 10.1155/2022/6151684

**Published:** 2022-10-10

**Authors:** Tajudeen Olabisi Obafemi

**Affiliations:** Department of Biochemistry, Afe Babalola University, PMB 5454, Ado-Ekiti, Nigeria

## Abstract

Nephrotoxicity is usually characterized by inefficiency of the kidney, thereby causing disruptions to electrolyte balance and blood acidity. This study aimed to evaluate the effect of hesperidin and gallic acid on serum electrolytes and ion pumps in Wistar rats subjected to aluminum chloride (AlCl_3_)-induced nephrotoxicity. Thirty Wistar rats were randomly divided into six groups of five animals apiece. Group one served as the negative control and received distilled water while the study lasted. Animals in groups 2–4 received 100 mg/kg/day AlCl_3_ throughout the study. Animals in groups 3 and 4 were also administered 100 mg/kg/day gallic acid and 100 mg/kg/day hesperidin, respectively. Groups 5 and 6 were treated with 100 mg/kg/day gallic acid only and 100 mg/kg/day hesperidin only, respectively. Treatments were administered orally via gavage for 28 days with distilled water as the vehicle. Animals were sacrificed after which levels of potassium, calcium, magnesium, phosphate, chloride, and bicarbonate ions were evaluated in the serum, while activities of Na^+^/K^+^ and Ca^2+^/Mg^2+^ ATPases were determined in kidney homogenate. Results showed that AlCl_3_ significantly (*p* < 0.05) inhibited activities of Na^+^/K^+^ and Ca^2+^/Mg^2+^ ATPases in addition to increasing serum levels of potassium, calcium, phosphate, and chloride, with concomitant decrease in serum levels of magnesium and bicarbonate. However, coadministration of AlCl_3_ with either gallic acid or hesperidin ameliorated all the disruptions caused by AlCl_3_. It could be concluded that gallic acid and hesperidin could be relevant in managing electrolyte imbalances and acidosis occasioned by kidney dysfunction.

## 1. Introduction

Nephrotoxicity is a condition in which detoxification and excretion functions of the kidney are impaired as a result of damage or destruction to the kidney by toxicants or drugs. Nephrotoxicity is usually implicated in the aetiology of acute kidney injury (AKI) [1]. Acute kidney injury is a health challenge of global dimension, annually affecting about 13.3% of people worldwide. AKI is in turn a risk factor for chronic kidney disease (CKD) [2, 3]. Both experimental and clinical data support the existence of a bidirectional relationship between AKI and CKD [4]. Due to the critical role of the kidney in maintenance of homeostasis, detoxification, and excretion of both drugs and toxic metabolites, the kidney is apparently a major target organ for toxicants [5].

In the course of daily interaction with the environment, humans and animals get exposed to various chemicals and heavy metals, which have the tendency to bioaccumulate in body tissues. Among the metallic elements in the Earth crust, aluminum has the third highest occurrence [6]. Exposure to aluminum mostly occurs via several of its compounds including aluminum chloride [7]. Sources of exposure of humans to aluminum include common household products such as shampoo, water treatment products, wood preservation products, food additives, and toothpaste. Nonhousehold sources of aluminum include particulate matters from cement factories and waste waters from industries [6, 7]. In spite of its low gastrointestinal absorption rate, aluminum has the tendency to accrue in essential organs such as the brain, kidney, and liver, and it over time causes cytotoxicity [8]. Moreover, the aluminum level has been reported to increase in both tissues and organs, with age [9]. An important reason for aluminum bioaccumulation is protein binding that constrains its ultrafiltration [9, 10]. Due to its limited excretion, primarily via urine, high doses of aluminum may lead to its renal retention and cause nephrotoxicity [11].

Maintenance of electrolyte levels within normal ranges is important for proper functioning of organs as well as several metabolic processes. The kidneys play an important role in maintaining electrolyte homeostasis. Thus, diseases and dysfunctions affecting the kidneys disturb their regulatory functions, thereby leading to imbalances in electrolyte level, with attendant life-threatening implications [12].

Hesperidin belongs to the flavanone class of flavonoids. Its antioxidant and anti-inflammatory effects in acute renal damage were earlier reported [13]. Gallic acid (3, 4, 5-trihydroxy benzoic acid) is a phenolic acid that has protective effect against oxidative stress-induced damage in tissues. Moreover, an earlier study reported its ability to prevent nephrotoxicity [14, 15]. A search through literature suggests that aluminum chloride is more often studied for its neurotoxicity compared with its nephrotoxicity. This study aimed to study and aimed to evaluate the protective effect of gallic acid and hesperidin, which are phytochemicals with proven pharmacological effects, against electrolyte imbalance in AlCl_3_-induced nephrotoxicity in Wistar rats.

## 2. Materials and Methods

### 2.1. Chemicals

Hesperidin and gallic acid were obtained from SantaCruz Biotechnology Inc, Heidelberg Germany. Kits for evaluating serum levels of potassium, calcium, and magnesium were obtained from Atlas Medical, Blankenfelde-Mahlow, Berlin, Germany. Kits for estimating serum levels of phosphate, chloride, and bicarbonate were purchased from Teco Diagnostics, Anaheim, USA. ELISA kits for evaluating Na^+^/K^+^ ATPase and Ca^2+^/Mg^2+^ ATPase were purchased from MyBiosource, Inc, San Diego, USA. All other chemicals and reagents used were of analytical grade.

### 2.2. Experimental Design

Thirty male rats of Wistar strain (180–200 g) were obtained from the animal research facility of Afe Babalola University, Ado-Ekiti, Nigeria. They were allowed to acclimatize for 10 days under standard conditions before commencement of the study. The experimental animals were randomly distributed into six groups (*n* = 5). Group 1 was the control and was administered distilled water only throughout the study. Animals in groups 2–4 were administered 100 mg/kg AlCl_3_ while the study lasted. Animals in groups 3 and 4 also received 100 mg/kg gallic acid and 100 mg/kg hesperidin, respectively, in addition to AlCl_3_. Groups 5 and 6 animals received 100 mg/kg gallic acid only and 100 mg/kg hesperidin only, respectively. All treatments were orally administered. Experimental animals were allowed access to food and water without restriction throughout the acclimatization and study periods. Animals were treated for 28 days. Doses of AlCl_3_, gallic acid, and hesperidin used in this study were as reported in previous studies [14, 16, 17].

### 2.3. Sample Preparation

Experimental animals were sacrificed under mild anaesthesia with diethyl ether 24 h after administration of the last doses of treatment. Blood was obtained via cardiac puncture and spun for 5 min at 3000 rpm to obtain serum for evaluating levels of potassium, calcium, phosphate, magnesium, chloride, and bicarbonate. Kidneys were dissected out and trimmed of excess fat, after which they were rinsed in isotonic saline. The kidneys were homogenized in chilled 50 mmol/l Tris-HCl buffer (pH 7.4). The homogenates were centrifuged at 3000 rpm for 10 min at 4°C after which the supernatant was used for evaluation of activities of Na^+^/k^+^ ATPase and Ca^2+^/Mg^2+^ ATPase. All animal studies complied with the Principle of Laboratory Animal Care [18]. Ethical approval (No : ABUAD/COS/2022/023) was obtained for the study from the Ethical Committee of the Afe Babalola University Research Directorate.

### 2.4. Biochemical Analyses

Serum levels of potassium, sodium, calcium, magnesium, phosphate, chloride, and bicarbonate were estimated spectrophotometrically by following instructions provided by kit manufacturers. Activities of Na^+^/K^+^ ATPase and Ca^2+^/Mg^2+^ were evaluated using ELISA according to kit manufacturers' instructions.

### 2.5. Statistical Analyses

Data were expressed as mean ± standard deviation. Data was analyzed with one-way ANOVA using using Graphpad prism 5 software. Mean comparison was done with the Tukey test. The level of statistical significance was held at *p* < 0.05.

## 3. Results

**Figure 1:**
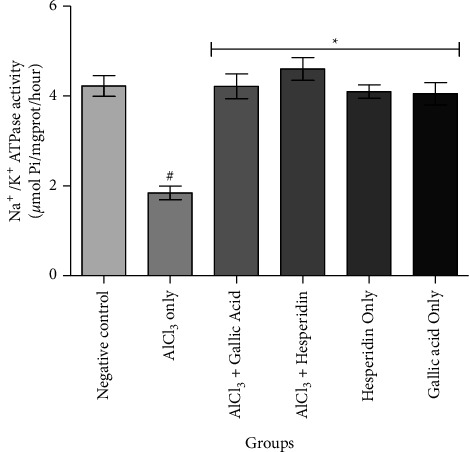
Effect of gallic acid and hesperidin on renal Na^+^/K^+^ ATPase activity in AlCl_3_-induced nephrotoxicity in Wistar rats. Values are expressed as the mean ± standard deviation of six determinations. ^#^*p* < 0.05 vs negative control, ^*∗*^*p* < 0.05 vs. AlCl_3_ only.

**Figure 2:**
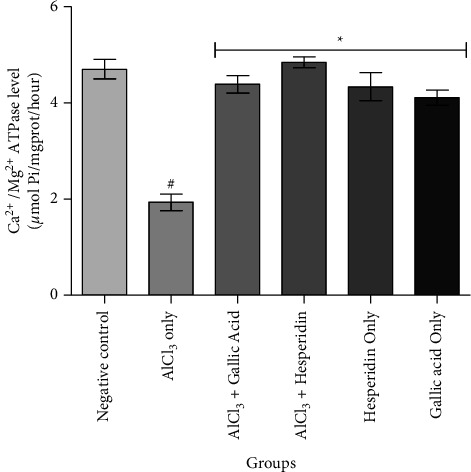
Effect of gallic acid and hesperidin on renal Ca^2+^/Mg^2+^ ATPase activity in AlCl_3_-induced nephrotoxicity in Wistar rats. Values are expressed as mean ± standard deviation of six determinations. ^#^*p* < 0.05 vs negative control, ^*∗*^*p* < 0.05 vs. AlCl_3_ only.

**Figure 3:**
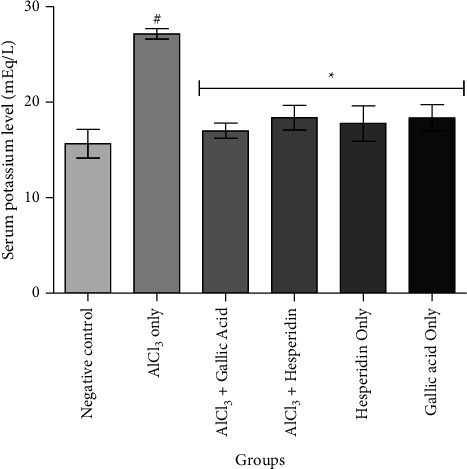
Effect of gallic acid and hesperidin on the serum potassium level in AlCl_3_-induced nephrotoxicity in Wistar rats. Values are expressed as the mean ± standard deviation of six determinations. ^#^*p* < 0.05 vs negative control, ^*∗*^*p* < 0.05 vs. AlCl_3_ only.

**Figure 4:**
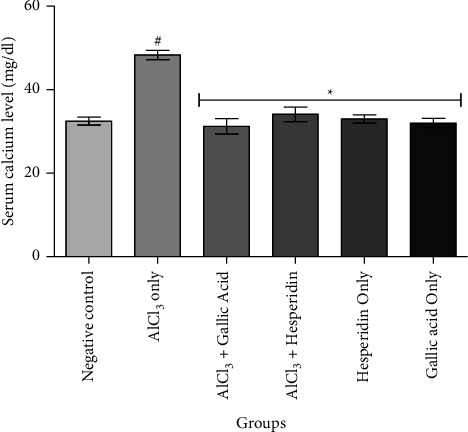
Effect of gallic acid and hesperidin on the serum calcium level in AlCl_3_-induced nephrotoxicity in Wistar rats. Values are expressed as the mean ± standard deviation of six determinations. ^#^*p* < 0.05 vs negative control, ^*∗*^*p* < 0.05 vs. AlCl_3_ only.

**Figure 5:**
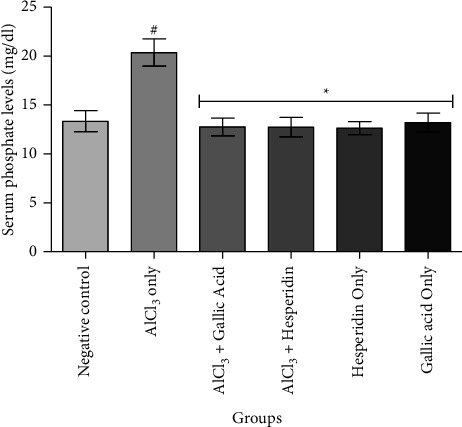
Effect of gallic acid and hesperidin on the serum phosphate level in AlCl_3_-induced nephrotoxicity in Wistar rats. Values are expressed as the mean ± standard deviation of six determinations. ^#^*p* < 0.05 vs negative control, ^*∗*^*p* < 0.05 vs. AlCl_3_ only.

**Figure 6:**
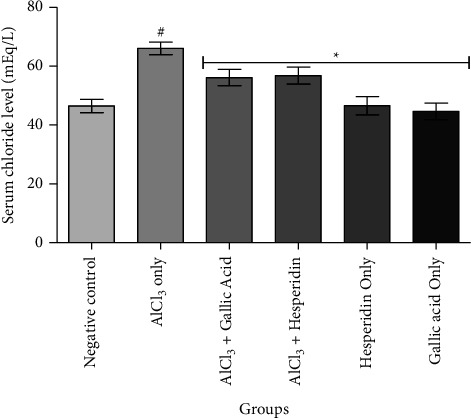
Effect of gallic acid and hesperidin on the serum chloride level in AlCl_3_-induced nephrotoxicity in Wistar rats. Values are expressed as the mean ± standard deviation of six determinations. ^#^*p* < 0.05 vs negative control, ^*∗*^*p* < 0.05 vs. AlCl_3_ only.

**Figure 7:**
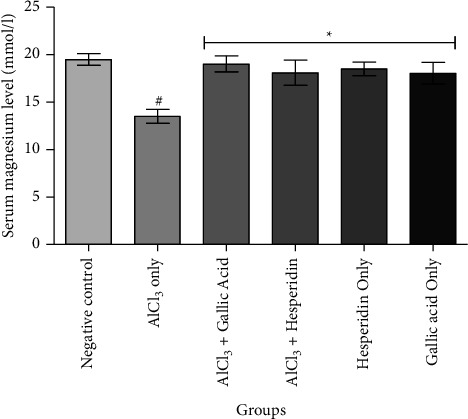
Effect of gallic acid and hesperidin on the serum magnesium level in AlCl_3_-induced nephrotoxicity in Wistar rats. Values are expressed as the mean ± standard deviation of six determinations. ^#^*p* < 0.05 vs negative control, ^*∗*^*p* < 0.05 vs. AlCl_3_ only.

**Figure 8:**
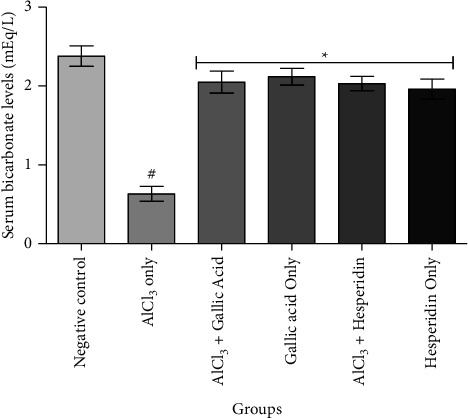
Effect of gallic acid and hesperidin on the serum bicarbonate level in AlCl_3_-induced nephrotoxicity in Wistar rats. Values are expressed as the mean ± standard deviation of six determinations. ^#^*p* < 0.05 vs negative control, ^*∗*^*p* < 0.05 vs. AlCl_3_ only.

Figure 1 shows that administration of AlCl_3_ only to rats occasioned a significant decrease in renal Na^+^/K^+^ ATPase activity while gallic acid and hesperidin prevented such decrease when they were coadministered with AlCl_3_. As presented in Figure 2, coadministration of AlCl_3_ with either gallic acid or hesperidin to experimental animals significantly (*p* < 0.05) increased Ca^2+^/Mg^2+^ ATPase activity, when compared with administration of AlCl_3_ only. In Figures 3–6, it was observed that administration of AlCl_3_ only to rats caused a significantly (*p* < 0.05) higher serum potassium, calcium, phosphate, and chloride levels, respectively. However, coadministration of gallic and hesperidin with AlCl_3_ prevented such an increase in all the electrolytes. Figures 7 and 8 showed that AlCl_3_ significantly (*p* < 0.05) lowered serum levels of magnesium and bicarbonate, respectively. This decrease was however prevented in rats administered with either gallic acid or hesperidin, in addition to AlCl_3_. Results from our study indicated that administration of gallic acid only and hesperidin only did not have adverse effects on the biochemical indices evaluated in this study.

## 4. Discussion

Nephrotoxicity is one of the commonest kidney disorders. It can be caused by several therapeutic and nontherapeutic chemicals that have toxic effects on various anatomical components of the kidney. These toxic effects eventually culminate in degeneration of morphology and function of the kidney [19]. Nephrotoxicity is capable of leading to both acute and chronic kidney diseases via tubular and glomerular damage [20]. Even though change in renal function, which is usually assessed by the glomerular filtration rate, blood urea nitrogen, serum creatinine, or urine output, is an important indication of nephrotoxicity, nephrotoxicants can cause kidney damage without causing alterations in these traditional biomarkers of kidney function [21]. *In vivo* and *in* vitro studies have established that aluminum is a prooxidant, whose basis of toxicity is oxidative stress and apoptosis, characterized by degeneration of the renal-tubular cells to [22, 23].

Electrolyte homeostasis is essential for proper functioning of numerous biological processes in the body. Consequently, dysfunctions of the kidney disrupt the electrolyte balance and may lead to life-threatening health conditions [24]. Na^+^/K^+^ ATPase and Ca^2+^/Mg^2+^ ATPase are enzymes found in all living organisms. They are involved in generating and maintaining ion gradients across biological membranes. Na^+^/K^+^ ATPase is highly expressed in the kidney, with the distal convoluted tubules having up to 50 million pumps per cell. The ion gradient generated by the enzyme is utilized by the kidney to filter the blood, maintain pH, and regulate levels of electrolytes in the blood [25]. Ca^2+^/Mg^2+^ ATPase is expressed in the basolateral membranes of the kidney where it contributes to the intracellular calcium concentration essential for kidney function and also contributes to maintenance of Ca^2+^ and Mg^2+^ homeostasis [26]. Chemical-induced inhibition of these enzymes might contribute to the disruption of electrolytes, a condition usually associated with nephrotoxicity and other kidney dysfunctions [12, 27]. Moreover, it has been reported that the action of Na^+^/K^+^ ATPase can be inhibited by aluminum in the kidney and liver, both *in vivo* and *in vitro* [24, 28]. In this study, it was observed that AlCl_3_ only significantly inhibited the activity of Na^+^/K^+^ ATPase and Ca^2+^/Mg^2+^ ATPase in the kidney of Wistar rats. However, when gallic acid and hesperidin were coadministered with AlCl_3_, inhibition of the enzymes was prevented. This observation is an indication that both gallic acid and hesperidin might be relevant in maintaining electrochemical gradients across membranes and thereby ameliorate imbalances in concentration of the electrolytes specific to these enzymes, thereby improving aluminum chloride-induced nephrotoxicity.

About 98% of potassium, which is the most abundant intracellular cation, is sequestered in the intracellular fluid. The electrochemical gradient of potassium is maintained by the action of Na^+^/K^+^ ATPase in the kidney [28]. Hyperkalemia is a serious health disorder that can lead to alterations in cardiac electrophysiology and ultimately death. Rise in serum levels of potassium is due to loss of nephron function and is an indication of deterioration of kidney functions [29]. In the present study, it was observed that AlCl_3_ caused hyperkalemia. However, coadministration of hesperidin and gallic acid reversed the high serum level observed in the group which administered AlCl_3_ only. This suggests that both gallic acid and hesperidin might be relevant in preventing cardiovascular events associated with impaired kidney function as hyperkalemia is an important link between both health conditions [30].

Calcium ion plays an important role in the regulation of several physiological processes including muscle contraction, secretory mechanisms, and excitation of neurons [31]. One of the causes of hypercalcemia is renal dysfunction characterized by decreased glomerular filtration and renal excretion. Hypercalcemia is usually associated with acute kidney disease [32, 33]. We report that AlCl_3_ caused a significant increase in serum calcium levels, a situation that was prevented with coadministration of AlCl_3_ with either gallic acid or hesperidin. It should however be noted that hypercalcemia arising from renal dysfunctions constitutes only a small fraction of all hypercalcemia cases [31, 33].

The kidneys play an important role in phosphorus homeostasis, and hence, renal dysfunctions can significantly disrupt phosphorus homeostasis. It was reported that the risk of death increases by 20% for every 1 mg/dl increase in serum phosphorus among CKD patients [34]. Even though phosphorus comes after calcium in order of abundance in the body, about 85% of phosphorus is found in bone and teeth as hydroxyapatite, while only 1% is located in the vascular space as inorganic phosphate. Hyperphosphatemia is predominantly observed in reduced renal functions, even though other probable causes exist [35]. Renal failure, presented as the reduced glomerular filtration rate, was reported to be the commonest cause of hyperphosphatemia [36]. In the present study, it was observed that the serum phosphate level was significantly higher in rats administered AlCl_3_ only. This is in contrast to the observation in rats coadministered AlCl_3_ with either gallic acid or hesperidin. Hyperphosphatemia increases the risk of mortality due to cardiovascular events in CKD patients mainly due to calcification of the vascular system [37]. Findings from this study suggest that gallic acid and hesperidin could prevent vascular calcification and its deleterious implications.

Magnesium is one of the most abundant intracellular divalent cations. It serves various essential roles in the body such as DNA synthesis, oxidative phosphorylation, cardiovascular tone, cofactor for enzymes, bone formation, and neuromuscular excitability [38]. Maintenance of the serum magnesium level is via an interaction between intestinal transport, renal transport, and bone exchange [32]. Thus, imbalances in the serum magnesium level are usually followed by serious clinical consequences. About of 95% of plasma magnesium is filtered by the glomerulus, followed by almost complete tubular reabsorption by the proximal and distal tubules and ascending loop of Henle. Thus, disruption in the tubular reabsorption processes could lead to hypomagnesemia as a result of increased renal loss [39]. In the present study, nephrotoxicity due to AlCl_3_ was observed to significantly lower the serum magnesium level in Wistar rats. Nevertheless, coadministration of AlCl_3_ with gallic acid and hesperidin to Wistar rats significantly increased the serum magnesium level, when compared with the AlCl_3_ only group. This observation implies that the ability of gallic acid and hesperidin to prevent hypomagnesemia could be due to improvement of tubular reabsorption of magnesium.

Chloride is a critical driver of several biological processes such as rennin secretion, renal sodium handling, blood pressure, and tubuloglomerular feedback [40]. A previous study identified an association between hyperchloremia and acute kidney injury [41], while another study correlated hyperchloremia with a worsened estimated glomerular filtration rate, which is an indication of nephrotoxicity [21, 40]. In our study, AlCl_3_ induced hyperchloremia in rats. However, gallic acid and hesperidin significantly lowered serum chloride levels when coadministered AlCl_3_. Our result suggests that gallic acid and hesperidin might have ameliorated the damaging effect of AlCl_3_ on the glomerular filtration rate in experimental animals.

In order to maintain regular acid-base balance, renal tubules reabsorb filtered HCO_3_^−^, in addition to synthesizing adequate HCO_3_^−^ in order to neutralize the intrinsic acid load [42]. The proximal convoluted tubule reabsorbs the bulk (80–85%) of filtered HCO_3_^−^, while the thick ascending limb of the loop of Henle reabsorbs the balance [43]. Failure of renal-tubular reabsorption of HCO_3_^−^ eventually leads to a reduction of serum bicarbonate concentration which ultimately contributes to metabolic acidosis [44]. Moreover, inability of residual nephrons to expel the daily acid load sequel to reduced renal mass is also known to lead to metabolic acidosis [45]. Both acute kidney injury and CKD are associated with metabolic acidosis [46]. In the present study, it was discovered that AlCl_3_ prompted metabolic acidosis by significantly lowering serum bicarbonate levels. Nonetheless, coadministration of gallic acid and hesperidin prevented metabolic acidosis possibly by preventing reduced renal mass and maintaining tubular reabsorption function of the kidneys.

## 5. Conclusion

This study corroborates the disruptive effect of aluminum on electrolyte homeostasis due to its nephrotoxic effects on renal functions. Apart from its effect on the serum levels of individual electrolytes, aluminum was also found to disrupt the activities of two enzymes involved in generation and maintenance of electrochemical gradients across membranes. Coadministration of gallic acid and hesperidin however prevented these biochemical alterations, an observation that further underlines the relevance of plant-derived bioactive compounds in health promotion. This study did not also discover a significant difference in the ability of gallic acid and hesperidin as far as maintenance of serum electrolyte levels was concerned.

## Data Availability

All data for this study are available on request.
